# Post-disaster building damage assessment based on improved U-Net

**DOI:** 10.1038/s41598-022-20114-w

**Published:** 2022-09-23

**Authors:** Liwei Deng, Yue Wang

**Affiliations:** Heilongjiang Provincial Key Laboratory of Complex Intelligent System and Integration, School of Automation, Harbin University of Science and Technology, Harbin, Heilongjiang China

**Keywords:** Environmental sciences, Natural hazards

## Abstract

When a severe natural disaster occurs, the extraction of post-disaster building damage information is one of the methods to quickly obtain disaster information. The increasingly mature high-resolution remote sensing technology provides a solid foundation for obtaining information about building damage. To address the issues with inaccurate building positioning in existing building damage assessment methods, as well as poor classification due to similar minor and major damage characteristics in building damage classification. Based on U-Net, we designed a two-stage building damage assessment network. The first stage is an independent U-Net focused on building segmentation, followed by a Siamese U-Net focused on building damage classification. The Extra Skip Connection and Asymmetric Convolution Block were used for enhancing the network's ability to segment buildings on different scales; Shuffle Attention directed the network's attention to the correlation of buildings before and after the disaster. The xBD dataset was used for training and testing in the study, and the overall performance was evaluated using a balanced F-score (F1). The improved network had an F1 of 0.8741 for localization and F1 of 0.7536 for classification. When compared to other methods, it achieved better overall performance for building damage assessment and was able to generalize to multiple disasters.

## Introduction

In recent decades, various natural disasters are frequently occurring all over the world. Earthquakes, floods, hurricanes, and other extremely destructive natural disasters not only cause massive property losses but also threaten the safety of human lives^1–3^. In addition to improving detection and early warning capability before a disaster occurs, it is critical to obtain disaster information quickly after a disaster occurs^4^.

The traditional method of obtaining real-time disaster assessment results is a manual on-site survey at the disaster site. Although the results of the manual processes are accurate, they are time-consuming and do not guarantee the safety of ground assessment personnel^5^. With the continuous growth of Remote Sensing (RS) technology, people can quickly get High-resolution Remote Sensing (HRS) images^6^ with much information. Machine learning (ML) and Deep Learning (DL) combined with HRS images to obtain post-disaster building damage information^7–9^. The existing methods for building damage assessment are generally based on post-disaster images or HRS images before and after the disasters^10–12^. It is common to use HRS images before and after a disaster, combined with change detection methods to detect building damage^13,14^.

In the beginning, researchers applied ML methods to the field of HRS images to assess the damage to buildings after a disaster. Support Vector Machines (SVM)^15,16^, Multilayer Feedforward Neural Network (MFNN)^17^, Linear Clustering (LC)^18^, and Random Forests (RF)^18^ have been used with some success for building damage assessment tasks. Li et al.^16^ used a kind of SVM method, based on hyperspectral data, experimented at pixel level and object level respectively, and obtained better results at object level. Cooner et al.^17^ Comparing several ML approaches to building damage assessment using pre-disaster and post-disaster HRS data from the 2010 Haiti earthquake, MFNN had the highest accuracy. Joshi et al.^18^ used aerial images after earthquake and tsunami, combined with the LC method to segment images till super-pixels, next extracted features, and used the RF as a classifier to assess the building damage level. Endo et al.^15^ proposed a moving window that aggregates adjacent pixels combined with SVM to detect building damage levels after a tsunami based on Synthetic Aperture Radar images. However, ML methods can only target building damage for one or two types of disasters. It is also necessary to design the feature extraction method according to the actual situation, and the robustness and generalization ability are poor.

DL methods compensate to some extent for the deficiencies of ML. The same model can be applied to multiple scenarios with little modification to achieve better results^10,19^. Some can achieve building damage assessment without supervision^20,21^. Ma et al.^19^ first used a Geographic Information System to extract post-disaster building damage information and delineate boundaries, and then using an improved convolutional neural network to classify the degree of damage to building groups. Tilon et al.^21^ developed an Unsupervised DL method for identifying post-disaster building damage; the Generating Adversarial Network was used for anomaly detection; only the image of the building in its undamaged state is required to output different types of damage classification and building location. Abdi et al.^10^ proposed a multimodal integration structure that combined orthophoto and off-bottom images to do post-earthquake building damage detection, based on deep transfer learning and multi-feature fusion methods. Li et al.^20^ proposed an unsupervised method to assess post-disaster damaged buildings, based on a set of generative adversarial networks, combined with a self-attention module, using unlabeled datasets to achieve better results.

Two issues need to be solved simultaneously in building damage assessment: building location and classification of building damage. Although the above deep learning methods have good results in robustness and generalization, it is difficult for end-to-end or unsupervised methods to achieve excellent performance in both building localization and building damage classification. In HRS images, the background of buildings is complex, the density of buildings is quite different. Furthermore, some buildings occupy fewer pixels in the images. when classifying building damage levels in building damage assessment, the characteristics of light damage and heavy damage are similar, making model prediction difficult. For this reason, we designed a two-stage building damage assessment model that focuses on building localization and building damage classification, respectively. The ACB and ESC modules were added to the model to enhance the model's ability to distinguish foreground–background while improving the accuracy of building segmentation at different scales. The SA module was used to make the model pay more attention to the correlation between HRS images before and after disasters, to optimize the classification ability of similar feature categories.

The contributions of this study are summarized as follows:

- Designed a two-stage building damage assessment network based on HRS images pre-disaster and post-disaster, localization stage and classification stage;
- Introducing ACB and ESC modules to enhance the accuracy of the building localization phase;
- Introducing the SA module with both channel and spatial attention into the model to enhance the performance of the classification phase.

The main purpose of this paper is to detect the damage level of buildings in different areas post-disaster more accurately and providing a valuable model for post-disaster relief. The main structure of this research is as follows, “Methods” section presents the information of the whole model, “Experiment and results” section presents the experimental results and data, “Discussion” section presents the experimental discussion, and “Conclusions” section describes the conclusion.

## Methods

### Building damage assessment framework

In the paper, we designed a two-stage network to solve the issue of building damage assessment, which included building detection and grading of building damage. The first stage primarily solved the problem of building detection, using the semantic segmentation method to segment the buildings. The second stage primarily solved the problem of building damage classification by drawing on the method of change detection, and using the first stage's building segmentation network as the foundation for creating Siamese Networks to classify building damage into four levels.

The U-Net^22^ involves two parts, an symmetric encoder and decoder, and the overall resemblance to a U-shaped structure. The encoder is responsible for extracting image features; the decoder is responsible for restoring the feature map to the original size; it has the advantage of fewer parameters and uncomplicated structure, which allows the network to be modified according to actual needs. The HRS images have abundant feature information and more complex building and background information, so U-Net is more suitable as the baseline model for this study than other semantic segmentation networks.

The first stage is the building segmentation network, and the U-Net as the baseline. Because the pre-disaster building features are intact and can better segment the location of the building, the pre-disaster images are used as the network input. The second stage is the network of building damage classification network, which is designed using the Siamese network, and is based on the building segmentation network. The network input is pre-disaster and post-disaster images, the network is initialized using the building segmentation weight, and the weights are shared in the encoder part.

**Figure 1:**
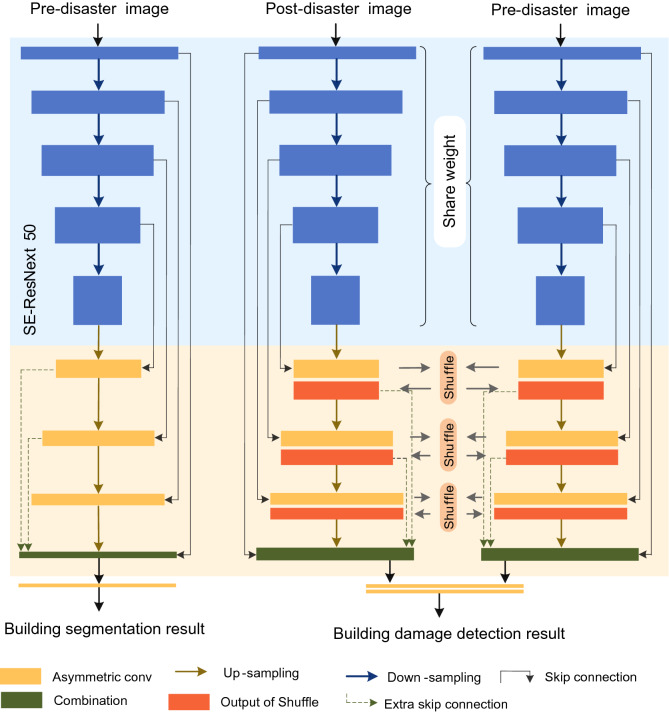
The building damage assessment framework. On the left is the building detection model; on the right is the building damage level classification model.

The main improvements of the network are the addition of Extra Skip Connection (ESC) in the decoder part, the replacement of partial convolution with Asymmetric Convolution Block (ACB)^23^, and the inclusion of a Shuffle Attention (SA)^24^ module in the decoder part of the Siamese Network. Figure 1 shows the framework for assessing building damage. Next, ACB, ESC, and SA attention will be introduced in detail.

### Backbone

Using SE-ResNeXt-50 as the backbone network of U-Net. SE-ResNeXt-50 adds the Squeeze-and-Excitation (SE)^25^ attention module and grouped convolution to the classic residual network (ResNet)^26^.

**Figure 2:**
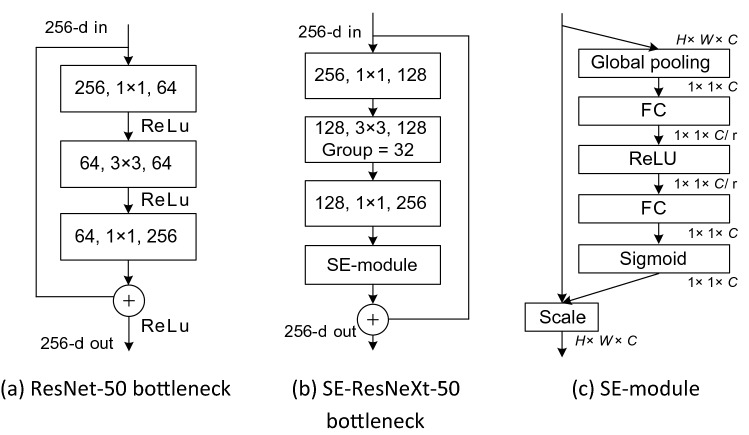
ResNet-50 and SE-ResNeXt-50 bottleneck blocks.

ResNet proposes the idea of residuals and bottleneck blocks, as is shown in Fig. 2a. Summing the output and input of the bottleneck block, avoiding vanishing gradient and explosion gradient; sing a combination of 1 × 1, 3 × 3, and 1 × 1 convolutions, the two 1 × 1 convolutions perform dimensionality reduction and dimensionality enhancement operations on the feature map, reducing the overall computational effort. ResNeXt^27^ proposes the idea of grouped convolution, which divides the input channels into 32 groups and uses parallel stacking of identical topological blocks instead of the original three-layer convolution, without significantly increasing the number of parameters and improving the accuracy of the model. The SE attention is channel attention that consists of two parts: squeeze and excitation. The squeeze is a 1 × 1 × *C* feature map obtained using global average pooling; excitation is a nonlinear transformation of the feature maps using a 1 × 1 convolution; the output is multiplied by the input so that the network strengthens the important features and suppresses others, as shown in Fig. 2c.

The SE-ResNeXt-50 combines the advantages of ResNeXt and SE, replacing the regular convolution with group convolution and adding SE at the end of bottleneck block to improve the ability of network features extraction, the bottleneck block of SE-ResNeXt-50 is shown in Fig. 2b. In this study, layers 1–4 of SE-ResNeXt-50 are used as layers 2–5 of the encoding, corresponding to 4 down-sampling of U-Net.

### Asymmetric convolution block

The similarity of building and background features in the HRS images causes the model to predict the background as buildings, which we replace the decoded layers 6–9 partial convolutions with ACB.

Standard convolution uses square convolution kernels of $a \times a$ size. The $a \times 1$ and $1 \times a$ convolution kernels are used instead of $a \times a$ convolution kernels in some studies, but this substitution is not equivalent. Although effective in reducing the number of parameters, some image features are inevitably lost, leading to model performance degradation. The $a \times a$, $a \times 1$ and $1 \times a$ convolution kernels of 2D convolution have additivity when the input and step sizes are the same, and the three different sized convolution kernels can share a sliding window.

1 $$I * X^{a \times a} + I * X^{a \times 1} + I * X^{1 \times a} = I * (X^{a \times a} \oplus X^{a \times 1} \oplus X^{1 \times a} )$$

where $I$ is the feature map; $X^{a \times 1}$ and $X^{1 \times a}$ are two kernels whose sizes can be compatible on $X^{a \times a}$; $\oplus$ is summed by the element in the corresponding position. Making use of this feature, ACB equivalently fuses convolutions of different sizes into the standard convolution. The horizontal and vertical kernels in asymmetric convolution form the kernel skeleton to obtain crossed receptive fields, enhance the extraction of useful information by the convolutional kernels, and suppress redundant information.

**Figure 3:**
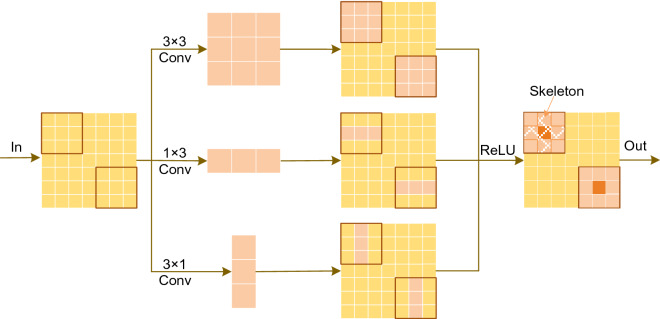
Asymmetric convolution block.

In this study, the 3 × 3, 3 × 1 and 1 × 3 sized convolutional kernels are used, as shown in Fig. 3. Three convolutional kernels acquire features in parallel, sum up the output feature maps and finally enhance their nonlinearity using the ReLU activation function. The 3 × 3 convolution captures the features in the fixed receptive field, and the 3 × 1 and 1 × 3 convolution kernels can enhance the features within the fixed receptive area from two dimensions during training. U-Net goes through two convolutional layers for each up-sampling.

The first convolutions are in charge of down-scaling the features from the prior layer; the second convolutions are in charge of splicing the encoder and prior layer features; modifying the second convolution to ACB.

### Extra skip connection

**Figure 4:**
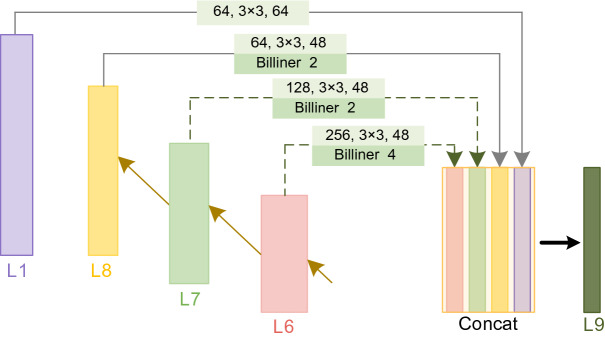
Extra skip connection.

The scale of buildings in HRS images varies greatly, and the model is hard to predict buildings with large scale differences accurately. Therefore, adding ESC to the decoder part of U-Net enable the model to fuse features of multiple scales, thereby increasing the segmentation accuracy of the model. The specific structure of ESC is shown in Fig. 4.

The low-level features by made of less convolution, less semantic information and more noise, but high-resolution location information and detail features are provided. The high-level features undergo more convolution, have lower resolution, and are less sensitive to location and detail features, but contain more robust semantic information. Combining semantic information from higher and lower levels can be called multi-scale feature fusion, generally classified into two types: parallel multi-branching structure and serial multi-branching structure. Inception modules, Atrous Spatial Pyramid Pooling^28^ modules that use atrous convolution, and Pyramid Pooling Module^29^ modules that use different size of pooling are the most well-known parallel multi-branch structures. Fully Convolutional Networks^30^ and U-Net represent the serial multi-branching architecture, and feature combinations are achieved by hopping layer connections.

This study draws inspiration from U-Net3+^31^, which combines high-level semantics and low-level details in different scale features through full-scale skip connection. Using serial multi-branching structures, layers 6–8 feature maps and feature maps from layer 1 are stitched together, and the number of connected feature maps after channels are reduced from 208 to 48 by 1 × 1 convolution to obtain layer 9.

Layers 6–8 are up-sampling using bilinear interpolation to make the feature map have the same dimension as layer 9, facilitating subsequent use of “Concat” to connect the feature maps. The number of channels is reduced to 48 by 3 × 3 convolution after up-sampling in layers 6–8. Due to the high number of channels in the layer 5, the computation is quite large even using 1 × 1 convolution, so the layer 5 is not connected.

### Shuffle attention

In the damage assessment of buildings in HRS images, a Siamese Network is built using U-Net. The HRS images before and after disasters are used as input to detect the damage degree of buildings using the change detection method. The features in the input pairs of images in change detection have some correlation, including unchanged backgrounds and buildings with varying degrees of damage. We add the SA module to notice this correlation.

Attention is generally divided into two categories: channel and spatial attention. SE module is typical channel attention. Typical spatial channel attention is Convolutional Block Attention Module^32^. Although the model gets better performance by combining the advantages of the channel and spatial attention, it necessarily increases the computational effort.

**Figure 5:**
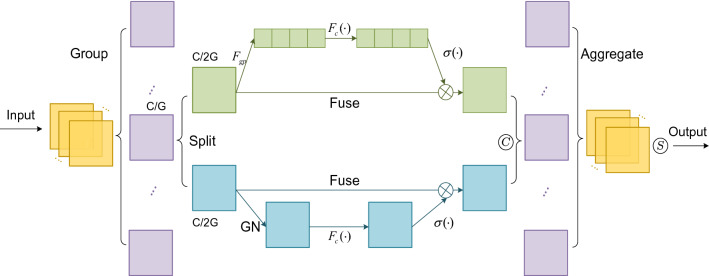
Shuffle attention.

The SA attention combines spatial and channel attention, which has the advantage of the small amount of calculation. The number of channels of the input feature maps $I \in {\mathbb{R}}^{C \times H \times W}$ is divided into $G$ groups, that is $I = [I_{1} ,I_{2} \cdots ,I_{G} ], \, I_{k} \in {\mathbb{R}}^{C/G \times H \times W}$. Before entering attention, the feature channel $I_{k}$ is again divided into two branches, both $I_{k1}$ and $I_{k2}$. One branch carries out channel attention; another branch carries out spatial attention. The overall structure of SA attention is shown in Fig. 5.

The channel attention section uses global average pooling to generate global information. The result of pooling is $S \in {\mathbb{R}}^{C/2G \times 1 \times 1}$.

2 $$S = F_{gp} (I_{k1} ) = \frac{1}{H \times W}\sum\limits_{i = 1}^{H} {\sum\limits_{j = 1}^{W} {I_{k1} (i,j)} }$$

Based on the idea of a gating mechanism to design adaptive feature output function and Sigmoid is the activation function, the output of the final $I_{k1}$ branch is $I_{k1}^{^{\prime}}$.

3 $$I_{k1}^{^{\prime}} = \sigma (F_{c} (S)) \cdot I_{k1} = \sigma (W_{1} S + b_{1} ) \cdot I_{k1}$$

where $W_{1} \in {\mathbb{R}}^{C/2G \times 1 \times 1}$ and $b_{1} \in {\mathbb{R}}^{C/2G \times 1 \times 1}$ are used to translate and scale $S$.

The spatial attention uses Group Norm (GN)^33^ to obtain the spatial data of the $I_{k2}$ branch, and the adaptive feature output function is used to enhance the feature representation of the $I_{k2}$ branch, and the final output of the $I_{k2}$ branch is $I_{k2}^{^{\prime}}$.

4 $$I_{k2}^{^{\prime}} = \sigma (W_{2} \cdot GN(I_{k2} )S + b_{2} ) \cdot I_{k2}$$

The output results of both $I_{k1}$ and $I_{k2}$ branches are combined to get a feature map $I_{k}^{^{\prime}}$ with the same number of channels as the input, $I_{k}^{^{\prime}} = [I_{k1}^{^{\prime}} ,I_{k2}^{^{\prime}} ] \in {\mathbb{R}}^{C/G \times H \times W}$. After all the features are aggregated, cross-group features are exchanged by channel shuffle, and the dimension of the final output feature map is the same as that of the input feature map.

We embed SA attention into the Siamese Network. There are two U-Net networks in the Siamese Network, one is responsible for extracting pre-disaster image features, and another is responsible for extracting post-disaster image features. Therefore, the feature of corresponding layers of the two U-Net decodes is combined and fed into the SA attention module. Finally, the output is used as the input of the next layer of the two U-Net, as a way to enhance the correlation of the before and after disaster images. The specific location of the SA module in the network is shown in Fig. 1, and attention is added to layers 6–8 of the decoder.

### Data

The xBD dataset^34^ is used in this study, mainly for post-disaster building damage assessment. The xBD dataset including HRS images before and after a variety of natural disasters worldwide, with a ground sampling distance of 0.8 m. Each image is 1024 × 1024 in size and has labeled files of building polygons and damage levels. The dataset classifies the damage levels of buildings into four categories: no damaged, minor damaged, major damaged and destroyed, which correspond to 0–3 in the labels. Table 1 shows the specific damage levels and detailed descriptions^34^.

**Table 1:** Damage level and damage description.

Number	Damage level	Damage description
0	No damaged	No sign of water, structural or burn damage
1	Minor damaged	Water or volcanic flow around buildings, roof cracks
2	Major damaged	Some walls or roofs collapse and were surrounded by water
3	Destroyed	Charred, completely collapsed, wholly or partially covered with water, or disappeared

The complete dataset includes three files: Train, Tier3, and Test, 11,034 pairs of HRS images before and after the disaster, 850,736 buildings, and a total coverage area of 45,361.79 square kilometers. Table 2 shows the detailed data of each file in the dataset.

**Table 2:** The xBD data distribution and their respective annotation counts.

Distribution	Image	Graphics
Train + Tier3	18,336	632,228
Test	1866	109,724

## Experiment and results

### Loss function

In the building detection phase, the crucial task is to distinguish the building from the background accurately. With the large number of pixels occupied by the background in HRS images, there is the problem of uneven distribution of foreground and background. Use a combination of loss functions, including Dice loss^35^ and Focal loss^36^. Dice loss is mainly used in the binary semantic segmentation task. There is an adjustable balance factor $\alpha$ in Focal loss. Since the ratio of building to a background in segmentation is about 1:9, the parameter $\alpha$ is set as 0.9 in the segmentation model, which can balance the sample difference. The Dice loss and Focal loss equations are as follows:

5 $$L_{Dice} = 1 - \frac{2pt + \varepsilon }{{p + t + \varepsilon }}$$

6 $$L_{Focal} = \left\{ {\begin{array}{*{20}c} { - \alpha (1 - p_{t} )^{2} \log (p_{t} )} \\ { - (1 - \alpha )(p_{t} )^{2} \log (1 - p_{t} )} \\ \end{array} } \right.\begin{array}{*{20}c} {,y = 1} \\ {,y = 0} \\ \end{array}$$

Among, $p$ is the predicted value; $\varepsilon$ is smooth to prevent the case of a zero denominator; $t$ is the label value; $p_{t}$ is the probability of a positive sample; $y$ is the label of the sample, $y = 1$ is the foreground and $y = 0$ is the background.

The main task completed in the building damage classification phase is the multi-classification task, in which a Cross-Entropy loss function for multi-classification is introduced on top of Dice loss and Focal loss. The Cross-Entropy loss formula is as follows:

7 $$L_{CE} = - \sum\limits_{e = 1}^{M} {y_{e} } \log (p_{e} )$$

where $M$ denotes the number of classifications; $y_{e}$ is a one-hot vector with elements taking only 0 and 1 values. The value 1 is taken if the category is the same as the category of the sample. Another is 0. $p_{e}$ denotes the probability that the predicted sample belongs to the class $e$.

In the xBD dataset, many buildings occupy fewer pixels and fall into the category of small targets. In the case of smaller targets, the Dice loss can produce severe oscillations. To balance this oscillation, we set weighted Focal loss in the segmentation stage, weighted Focal loss and Cross-Entropy loss in the classification stage. After several experiments and comparisons, the combination of loss functions with fewer oscillation occurrences is selected^37^.

### Metric

Building segmentation accuracy is evaluated using two evaluation indicators, $IOU$ and $F1$, during the building inspection phase. $F1$ also known as the balanced F-score, is the summed average of precision and recall, which combines the results of precision and recall. The comprehensive performance of the model can be effectively evaluated. The $IOU$ is called the intersection-merge ratio, which is the ratio of the intersection and merge of the model's predicted and actual values. The equations of $IOU$ and $F1_{loc}$ are as follows:

8 $$IOU = \frac{TP}{{TP + FP + FN}}$$

9 $$P = \frac{TP}{{TP + FP}}$$

10 $$R = \frac{TP}{{TP + FN}}$$

11 $$F1_{loc} = \frac{2}{1/P + 1/R} = \frac{2PR}{{P + R}}$$

where $P$ is the Precision; $R$ is the Recall; $TP$ is True Positive, the inference is true, the reality is true; $FP$ is False Positive, the inference is true, the reality is false; $FN$ is False Negative, the inference is false, the reality is true; $TN$ is Ture Negative, the inference is false, the reality is false.

$F1$ is applied to evaluate the performance of the second stage model. In the study, the damage levels were divided into four categories, at which point the $F1$ scores needed some modification to become a summed average of the four categories $F1$ score. The $F1_{cls}$ formula is as follows:

12 $$F1_{cls} = \frac{4}{{\sum\limits_{i = 0}^{3} {\left( {\frac{1}{{F1_{cls}^{i} + \varepsilon }}} \right)} }}$$

For the overall evaluation metrics of the model, we refer to the Xview2 competition, with $F1_{loc}$ and $F1_{cls}$ weighted separately to obtain the final $F1_{overall}$.

13 $$F1_{overall} = 0.3 \times F1_{loc} + 0.7 \times F1_{cls}$$

Compared to the accurate segmentation of the building, the precise assessment of the damage level of the building is more important, so the classification part of the score has a greater weight.

### Training details

The two-stage model was trained using the AdamW optimizer, the model used Python 3.7 PyTorch, and the model was trained and tested on an NVIDIA Quadro RTX 6000 GPU. Enhancements to the data during the training phase include flipping, rotating, scaling, contrast, and elastic transformations. The original size of the input image was 1024 × 1024 and the resized ones after data enhancement are 512 × 512. The batch size for both training and testing was 4. The building segmentation phase was initialized with pre-trained weight from the PyTorch Library with a learning rate of $lr = 0.00015$ and 35 epochs of training; the building classification phase with $lr = 0.0002$ and 20 epochs of training; the whole training process was about 18–20 h.

### Results

**Figure 6:**
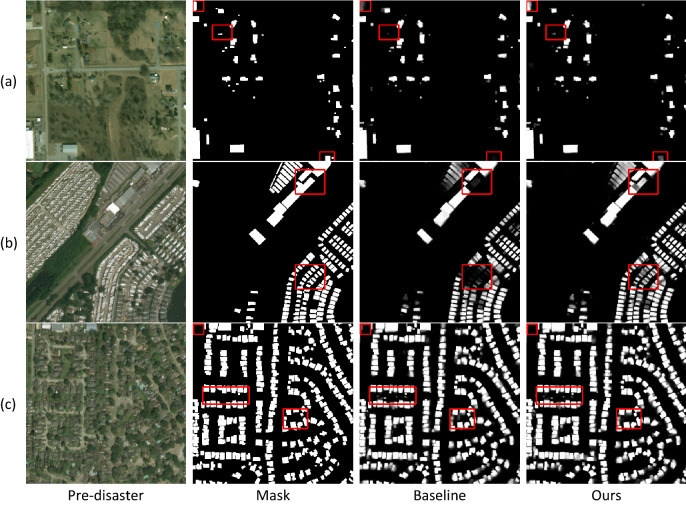
Visualization of building segmentation results.

The performance of the model was tested using a portion of the Test data provided by xBD data as a test set. Figure 6 shows the segmentation results for sparse, dense, and simultaneous inclusion of buildings with large-scale differences. From Fig. 6a, compared with the base-line model, the improved model can effectively segment sparse buildings and enhanced the model's perception of buildings at the edges of the images; Fig. 6b show that the model can provide better segmentation of buildings with large scale differences; Fig. 6c show that the model can extract dense buildings more accurately, although the edges of the buildings are slightly blurred.

In the building segmentation stage, four models were trained, including the baseline model, only the ESC, only the ACB, and the model with both "ESC + ACB". Table 3 shows the evaluation indexes between our model and other models.

**Table 3:** Evaluation of building segmentation results.

Model	$F1_{loc}$	$IOU$
xBD^34^	0.7900	–
Weber^38^	0.8350	–
RescueNet^39^	0.8400	–
Dual-HRNet^40^	0.8657	
Peng^41^	0.8380	0.7385
FCN^30^	0.8413	0.7468
Baseline	0.8617	0.7571
Baseline + ESC	0.8672	0.7655
Baseline + ACB	0.8736	0.7756
Baseline + ESC + ACB	**0.8741**	**0.7763**

Significant values are in bold.

From Table 3, in the segmentation stage, when used the same dataset, is compared with other studies, this model performs best. It could be seen that adding ESC or ACB alone in the decoding part of the model had some improved segmentation accuracy ($IOU{}_{ESC}$ improved by 0.0084; $IOU{}_{ACB}$ improved by 0.0185), and adding both modules at the same time finally yields $F1_{loc} = 0.8741$ and $IOU = 0.7763$.

Segmenting the buildings was only the first step. The next task was to classify the damage level of the buildings based on the segmented buildings. The SA attention was used to more aware of the changes in buildings before and after the disaster, and to improve the model's detection accuracy on level 1–2 damage. The final results of the building damage assessment are shown in Fig. 4, and the evaluation indexes used $F1_{overall}$, $F1_{loc}$ with a weight of 0.3 and $F1_{cls}$ with a weight of 0.7 as introduced in Metric. To verify the performance of the designed model, we compared the performance of several models using the xBD dataset, including the baseline model provided by the official at beginning of the competition, using the change detection method, and the method of image feature fusion before and after the disaster. The specific data comparison is shown in Table 4.

**Table 4:** Overall building damage assessment results.

Model	$F1_{overall}$	$F1_{loc}$	$F1_{cls}$	$F1_{cls}^{0}$	$F1_{cls}^{1}$	$F1_{cls}^{2}$	$F1_{cls}^{3}$
xBD^34^	0.2600	0.7900	0.0300	0.7211	0.0235	0.0105	0.4262
Weber^38^	0.7410	0.8350	0.6970	0.9060	0.4930	0.7220	0.8370
RescueNet^39^	0.7410	0.8400	0.7348	0.8832	0.5628	**0.7711**	0.8079
Dual-HRNet^40^	0.7678	0.8657	0.7257	–	–	–	–
Peng^41^	0.7464	0.8380	0.7071	0.8765	0.5129	0.7145	**0.8568**
FCN^30^	0.7584	0.8413	0.7228	0.9444	0.5243	0.7337	0.8297
Baseline	0.7764	0.8617	0.7398	0.9472	0.5658	0.7289	0.8251
Ours	**0.7898**	**0.8741**	**0.7536**	**0.9521**	**0.5778**	0.7538	0.8335

Significant values are in bold.

The official baseline model did not do the corresponding data enhancement according to the dataset's characteristics and obtained poor results in the classification stage. Weber et al.^38^ used change detection methods to extract pre-and post-disaster image features respectively and shared weights at the end to fuse the features. RescueNet^39^ was a single-stage network that fuses the pre-disaster and post-disaster to complete the segmentation and classification tasks simultaneously. Dual-HRNet^40^ was a model of building damage detection built based on HRNet. Peng et al.^41^ designed an end-to-end change detection model, which we applied to building damage assessment. The FCN^30^ semantic segmentation model was applied to the building damage assessment task with the same structure as the two-stage model, and only the location of UNet was changed to FCN.

Compared with the above models, the U-Net model used in this paper is based on a two-stage model, and the introduction of ESC and ACB in the building segmentation stage makes the model achieve better results in segmentation; as we integrate SA attention in the classification stage, it showed better results in the overall classification and each damage classification index. Although we did not obtain better results in heavy damage and complete damage than the other two models, our model achieved $F1_{loc} = 0.8741$ and $F1_{cls} = 0.7536$ in the overall segmentation and classification metrics, undamaged score $F1_{cls}^{0} = 0.9521$ and lightly damaged score $F1_{cls}^{1} = 0.5778$.

**Figure 7:**
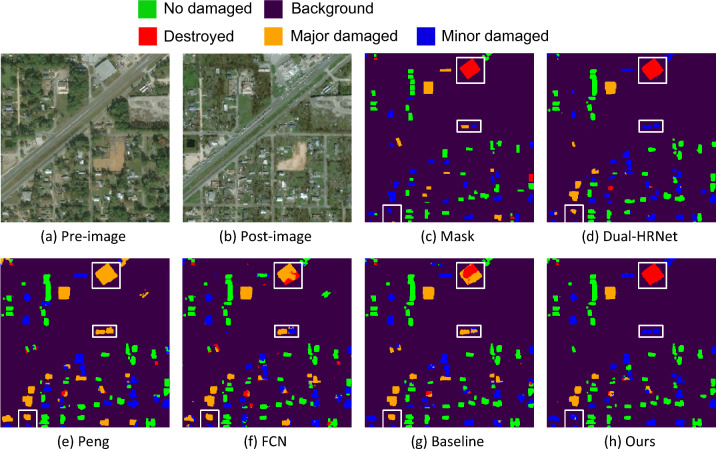
Classification of building damage in Hurricane-Michael.

**Figure 8:**
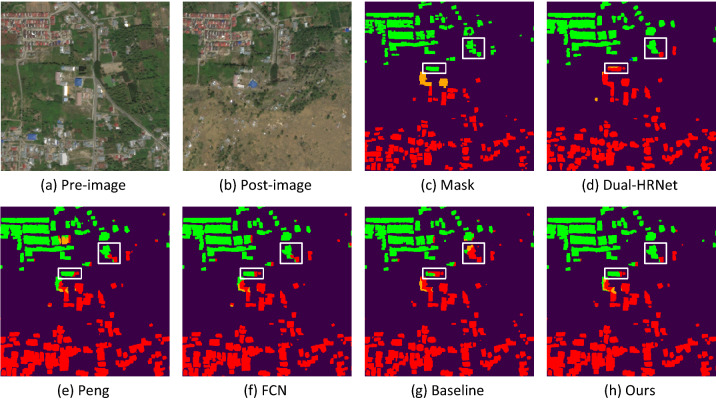
Classification of building damage in Palu-Tsunami.

**Figure 9:**
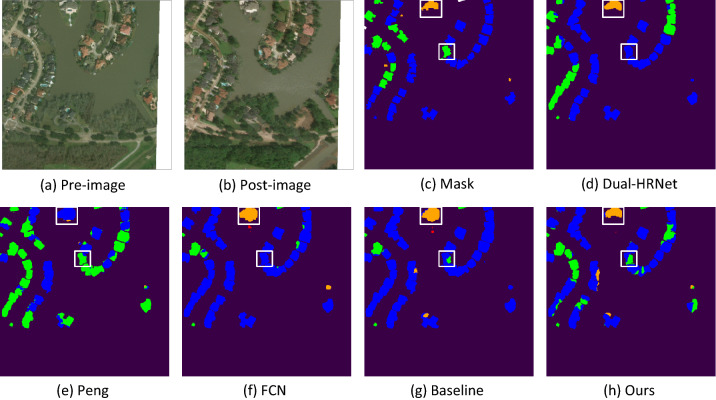
Classification of building damage in Hurricane-Harvey.

To gain a better understanding of the effect of model improvement, we selected typical samples from the test set to compare the results. These three samples are two hazards from three regions with different levels of building damage. Figures 7, 8 and 9 show the visualization results of the damage assessment from several models. From these figures, we can see that the end-to-end building change detection model (model of Peng et al.^41^) considers only the change of building location. However, the building location is constant in the building damage assessment, and only the roof of the building is changing, so many buildings are mis-segmented and with blurred contours. When FCN is used as the baseline network of the two-stage model, the building damage classification results are also poor because FCN cannot retain more semantic information in the up-sampling part. Although the Dual-HRNet model has a better detection effect, the model is large, takes up too much memory during training, and takes a long time to predict.

Figure 7 mainly shows the predicted results for Hurricane-Michael, with the detection results in the presence of all four types of damage. The majority of the intact buildings are correctly classified. The buildings in the lower left of the figure are mostly undamaged or lightly damaged, yet some lightly damaged buildings are still classified as heavily damaged. A larger building in the upper right corner of the figure is completely damaged, and the baseline model initially classified it as heavily damaged, but the improved model is correctly classified. By comparing the images before and after the disaster, it can be seen that the building will inevitably be offset to some extent due to different angles of shooting, while there are more plants around the building to cover the building, and most of the plants are broken after the disaster, and some of them are fused with the damaged part of the building, all the above factors may lead to the misclassification of similar damage. After the model improvement, minor damage and major damage have some enhancements.

Figure 8 shows mainly the predicted results of Palu-Tsunami, primarily to observe the detection results of complete damage and undamaged. The classification results in the more continuous intact and completely damaged are more accurate, and there are misclassifications in the classification results of the positions adjacent to the two categories. Two major damaged buildings were misclassified in the middle of the figure, and the two images pre-disaster and post-disaster were overlaid and compared. The completely damaged buildings were all covered with sediment, and the two buildings in the middle were severely displaced. The positions of the buildings were determined by the pre-disaster images. The positions of the buildings were severely shifted after the disaster, but the buildings were not completely damaged. The improved classification result of completely undamaged is more accurate. Figure 9 is mainly for observing the detection of minor damage to buildings, and the model has the lowest detection accuracy for minor damage compared to several other damage classes. The figure shows that the improved model has improved the detection of minor damage, but there are still some mixed cases of minor damage and undamaged categories. On the one hand, there are few data of minor damaged buildings; on the other hand, the characteristics of minor damaged buildings are not outstanding, so they can be easily misclassified as undamaged.

To better illustrate the specific effects of each module introduced on the model, we did some ablation experiments on each module. The ablation results were shown in Table 5.

**Table 5:** Results of ablation experiments for building damage assessment.

Model	$F1_{overall}$	$F1_{loc}$	$F1_{cls}$	$F1_{cls}^{0}$	$F1_{cls}^{1}$	$F1_{cls}^{2}$	$F1_{cls}^{3}$
Baseline	0.7764	0.8617	0.7398	0.9472	0.5658	0.7289	0.8251
Baseline + ESC	0.7794	0.8672	0.7418	0.9503	0.5528	0.7424	**0.8445**
Baseline + ACB	0.7841	0.8736	0.7457	0.9509	0.5721	0.7408	0.8234
Baseline + ESC + ACB + SA (ours)	**0.7898**	**0.8741**	**0.7536**	**0.9521**	**0.5778**	**0.7538**	0.8335

Significant values are in bold.

### ESC and ACB

After ESC and ACB are added to the network, the building segmentation index is improved by 1.24% compared with the baseline, which improves the segmentation ability of dense buildings and multi-scale buildings.

### SA

After adding SA attention to the model, the overall performance of the classification stage is improved, and the building damage classification index is increased by 1.38%. The building damage assessment index reaches $F1_{overall} = 0.7536$. In the classification of building damage. The model after adding SA has no damaged score of $F1_{cls}^{0} = 0.9521$, a minor damaged score of $F1_{cls}^{1} = 0.5778$, and a major damaged score of $F1_{cls}^{2} = 0.7538$, all are the highest. The model with SA attention outperforms the model without SA attention.

## Discussion

Building damage assessment tasks include building localization and building dam-age classification. For this purpose, the main work of this paper is to design and improve a two-stage building damage assessment network based on U-Net.

The first stage corresponds to the building localization task. Combining the idea of asymmetric convolution, the standard convolution in up-sampling is replaced with ACB to enhance the ability to distinguish foreground and background when the network segments buildings. Using the idea of better retention of small-scale features at shallow levels and better retention of large-scale features at high levels, ESC is designed to fuse different scale features and enhance the segmentation ac-curacy of the network for multi-scale buildings. A fair comparison was made between other methods and our method on the same test set, and for an objective assessment of the building semantic segmentation results $IOU$ and $F1$ metrics are used. As shown in Table 3, both metrics are better than the other methods. As shown in Fig. 6, when the buildings are sparse, it can segment the buildings at the edge of the image better; when the buildings in the image have different scales, and the image has similar information as the buildings, it can still segment the buildings accurately; when the buildings are dense, it can segment the single buildings better, and very few adhesions occur. The edges of the building appear vignetted when there is more shading around the building. Overall ACB and ESC both enhance the segmentation effect of the building.

The second stage uses the change detection approach to complete the building damage classification task by combining two first-stage building segmentation networks into a Siamese Network. In order to make the change detection network cost more attention to the changes in building information in remote sensing images before and after the disaster, this paper embeds the SA attention by incorporating the grouping idea into the up-sampling middle of the Siamese network, which enhances the performance of building damage classification. The $F1$ index is mainly used in the classification stage, and different weights are assigned to localization and classification respectively in the overall evaluation, as shown in Eq. 13. As shown in Fig. 7, when all four damage classes are present in the image, undamaged and complete damage are mostly classified correctly, and there are some misclassifications in light and heavy damage, but the misclassifications are improved after embedding SA attention. As shown in Fig. 8, when there is a large area of undamaged and total damage, the junction of the two types of damage is misclassified, and the improved network has fewer misclassifications. As shown in Fig. 9, the improved model has significantly reduced the misclassification of minor damage. However, there are still a large number of false predictions in the parts adjacent to undamaged and minor damage. These improvements were also reflected in the evaluation metrics, as shown in Table 4, where our net-work was better than the other networks in the overall damage classification metrics, un-damaged and lightly damaged; the metrics for lightly damaged, although the highest, were much lower than the other categories, probably due to the lack of data for lightly damaged. Although there is a slight lack of severe damage and total damage, they all reach a good level.

The improved two-stage building damage detection network has good robustness in a variety of natural hazards, and two of the more common ones are shown in Figs. 7 and 8. The network designed in this paper achieves good prediction results in building damage assessment, and in subsequent studies will continue to address two remaining problems, one is segmentation in the presence of building occlusion, and the other is that the index of minor building damage is much lower than other categories.

## Conclusions

In this research, we designed a network for building damage detection, which could segment buildings while classifying the damage level of buildings after a disaster. By quantifying the impact of each added module on the model performance through ablation experiments. In the segmentation stage, ESC improves the model's ability to segment multi-scale buildings; ACB improves the model's foreground and background segmentation performance; and ESC and ACB improve the model's ability to segment complex buildings. In the classification stage, SA attention It enables the network to pay attention to the correlation of RS images before and after the disaster, resulting in improved classification accuracy. When compared to the Dual-HRNet model, the positioning accuracy was improved by 0.84% and the classification accuracy was improved by 2.79%. Our model could be applied to multiple natural disasters, which helped in the application and generalization of the model. This work contributed to the integration and advancement of DL methods and RS technologies.

## Data Availability

All data included in this study are available upon request by contact with the corresponding author.

## References

[1] Deniz D, Arneson EE, Liel AB, Dashti S, Javernick-Will AN (2017). Flood loss models for residential buildings, based on the 2013 Colorado floods. Nat. Hazards.

[2] Du, Y., Gong, L., Li, Q. & Wu, F. Earthquake-induced building damage assessment on SAR multi-texture feature fusion. In *Proceedings of the IGARSS 2020—2020 IEEE International Geoscience and Remote Sensing Symposium.* 6608–6610 (2020).

[3] Lin C, Li Y, Liu Y, Wang X, Geng S (2021). Building damage assessment from post-hurricane imageries using unsupervised domain adaptation with enhanced feature discrimination. IEEE Trans. Geosci. Remote Sens..

[4] Naito S (2020). Building-damage detection method based on machine learning utilizing aerial photographs of the Kumamoto earthquake. Earthq. Spectra.

[5] Allali SA, Abed M, Mebarki A (2018). Post-earthquake assessment of buildings damage using fuzzy logic. Eng. Struct..

[6] Wang B, Lu X, Zheng X, Li X (2019). Semantic descriptions of high-resolution remote sensing images. IEEE Geosci. Remote Sens. Lett..

[7] Koshimura S, Moya L, Mas E, Bai Y (2020). Tsunami damage detection with remote sensing: A review. Geosciences.

[8] Sharma TPP (2019). Review of flood disaster studies in Nepal: A remote sensing perspective. Int. J. Disaster Risk Reduct..

[9] Zhao X (2021). Advances of satellite remote sensing technology in earthquake prediction. Nat. Hazard. Rev..

[10] Abdi G, Jabari S (2021). A multi-feature fusion using deep transfer learning for earthquake building damage detection. Can. J. Remote Sens..

[11] Li Y, Hu W, Dong H, Zhang X (2019). Building damage detection from post-event aerial imagery using single shot multibox detector. Appl. Sci..

[12] Nex F, Duarte D, Tonolo FG, Kerle N (2019). Structural building damage detection with deep learning: Assessment of a state-of-the-art CNN in operational conditions. Remote Sens..

[13] Asokan A, Anitha J (2019). Change detection techniques for remote sensing applications: A survey. Earth Sci. Inf..

[14] Chen H, Shi Z (2020). A spatial-temporal attention-based method and a new dataset for remote sensing image change detection. Remote Sens..

[15] Endo Y, Adriano B, Mas E, Koshimura S (2018). New insights into multiclass damage classification of tsunami-induced building damage from SAR images. Remote Sens..

[16] Li P, Xu H, Guo J (2010). Urban building damage detection from very high resolution imagery using OCSVM and spatial features. Int. J. Remote Sens..

[17] Cooner AJ, Shao Y, Campbell JB (2016). Detection of urban damage using remote sensing and machine learning algorithms: Revisiting the 2010 Haiti earthquake. Remote Sens..

[18] Joshi, A. R., Tarte, I., Suresh, S. & Koolagudi, S. G. Damage identification and assessment using image processing on post-disaster satellite imagery. In *Proceedings of the 2017 IEEE Global Humanitarian Technology Conference (GHTC).* 1–7 (2017).

[19] Ma H (2020). Improved CNN classification method for groups of buildings damaged by earthquake, based on high resolution remote sensing images. Remote Sens..

[20] Li Y (2020). Unsupervised domain adaptation with self-attention for post-disaster building damage detection. Neurocomputing.

[21] Tilon S, Nex F, Kerle N, Vosselman G (2020). Post-disaster building damage detection from earth observation imagery using unsupervised and transferable anomaly detecting generative adversarial networks. Remote Sens..

[22] Ronneberger, O., Fischer, P. & Brox, T. U-Net: Convolutional networks for biomedical image segmentation. In *Proceedings of the Medical Image Computing and Computer-Assisted Intervention—MICCAI 2015.* 234–241 (2015).

[23] Ding, X., Guo, Y., Ding, G. & Han, J. ACNet: Strengthening the kernel skeletons for powerful CNN via asymmetric convolution blocks. In *Proceedings of the IEEE/CVF International Conference on Computer Vision.* 1911–1920 (2019).

[24] Zhang, Q.-L. & Yang, Y.-B. SA-Net: Shuffle attention for deep convolutional neural networks. In *Proceedings of the ICASSP 2021–2021 IEEE International Conference on Acoustics, Speech and Signal Processing (ICASSP).* 2235–2239 (2021).

[25] Hu J, Shen L, Albanie S, Sun G, Wu E (2020). Squeeze-and-excitation networks. IEEE Trans. Pattern Anal. Mach. Intell..

[26] He, K., Zhang, X., Ren, S. & Sun, J. Deep residual learning for image recognition. In *Proceedings of the IEEE Conference on Computer Vision and Pattern Recognition.* 770–778 (2016).

[27] Xie, S., Girshick, R., Dollár, P., Tu, Z. & He, K. Aggregated residual transformations for deep neural networks. In *Proceedings of the IEEE Conference on Computer Vision and Pattern Recognition.* 1492–1500 (2017).

[28] He H, Yang D, Wang S, Wang S, Li Y (2019). Road extraction by using atrous spatial pyramid pooling integrated encoder-decoder network and structural similarity loss. Remote Sens..

[29] Lian X, Pang Y, Han J, Pan J (2021). Cascaded hierarchical atrous spatial pyramid pooling module for semantic segmentation. Pattern Recogn..

[30] Shelhamer E, Long J, Darrell T (2017). Fully convolutional networks for semantic segmentation. IEEE Trans. Pattern Anal. Mach. Intell..

[31] Huang, H. *et al.* Unet 3+: A full-scale connected unet for medical image segmentation. In *Proceedings of the ICASSP 2020–2020 IEEE International Conference on Acoustics, Speech and Signal Processing (ICASSP).* 1055–1059 (2020). 10.1109/ICASSP40776.2020.9053555 PMC7543994 33041676

[32] Woo, S., Park, J., Lee, J.-Y. & Kweon, I. S. CBAM: Convolutional block attention module. In *Proceedings of the European Conference on Computer Vision (ECCV).* 3–19 (2018).

[33] Wu, Y. & He, K. Group normalization. In *Proceedings of the European Conference on Computer Vision (ECCV).* 3–19 (2018).

[34] Gupta, R. *et al.* xBD: A dataset for assessing building damage from satellite imagery. Preprint at 10.48550/arXiv.1911.09296 (2019).

[35] Milletari, F., Navab, N. & Ahmadi, S. V-Net: Fully convolutional neural networks for volumetric medical image segmentation. In *Proceedings of the 2016 Fourth International Conference on 3D Vision (3DV).* 565–571 (2016).

[36] Lin, T.-Y., Goyal, P., Girshick, R., He, K. & Dollár, P. Focal loss for dense object detection. In *Proceedings of the Proceedings of the IEEE International Conference on Computer Vision.* 2980–2988 (2017).

[37] Zhu W (2019). AnatomyNet: Deep learning for fast and fully automated whole-volume segmentation of head and neck anatomy. Med. Phys..

[38] Weber, E. & Kané, H. Building disaster damage assessment in satellite imagery with multi-temporal fusion. Preprint at 10.48550/arXiv.2004.05525 (2020).

[39] Gupta, R. & Shah, M. RescueNet: Joint building segmentation and damage assessment from satellite imagery. In *Proceedings of the 2020 25th International Conference on Pattern Recognition (ICPR).* 4405–4411 (2021).

[40] Koo, J., Seo, J., Yoon, K. & Jeon, T. *Dual-HRNet for Building Localization and Damage Classification*. https://github.com/DIUx-xView/xView2_fifth_place/blob/master/figures/xView2_White_Paper_SI_Analytics.pdf (2019).

[41] Peng D, Zhang Y, Guan H (2019). End-to-end change detection for high resolution satellite images using improved UNet++. Remote Sens..

